# Elucidating the Molecular Mechanisms of Hederagenin-Regulated Mitophagy in Cervical Cancer SiHa Cells through an Integrative Approach Combining Proteomics and Advanced Network Association Algorithm

**DOI:** 10.1021/acs.jproteome.5c00022

**Published:** 2025-03-26

**Authors:** Hao Sun, Dan Wang, Yongquan Zheng, Yiqing Ye

**Affiliations:** † Pharmacy Department, Women’s Hospital, Zhejiang University School of Medicine, Hangzhou 310006, China; ‡ Pharmacy Department, Zhejiang Hospital, Hangzhou 310030, China

**Keywords:** hederagenin, cervical cancer, SiHa cells, mitophagy, TMT proteomics, network association algorithm

## Abstract

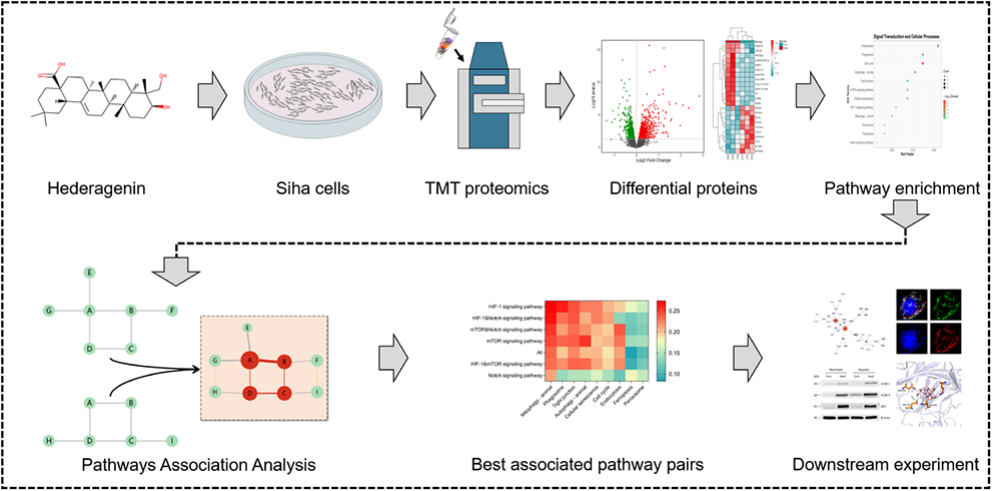

Hederagenin (Hed), a natural triterpenoid, exhibits antitumor potential in cervical cancer. The present study was designed to explore Hed’s regulatory mechanisms on mitophagy in SiHa cervical cancer cells, employing tandem mass tag (TMT) proteomics and an advanced network association algorithm (NAA). Our findings revealed that Hed decreased SiHa cell viability, induced apoptosis, and altered mitochondrial membrane potential. Notably, Hed inhibited mitophagic flux under both normoxic and hypoxic conditions. Through TMT proteomics analysis and innovative NAA, we identified a close association between the HIF-1 signaling pathway and mitophagy. Network analysis further suggested that Hed acts on a target network centered on SRC, STAT3, AKT1, and HIF1A. Western blot analysis confirmed the expression and phosphorylation status of these targets in response to Hed. This study elucidates the molecular mechanisms underlying Hed’s regulation of mitophagy in SiHa cells, offering novel insights and potential therapeutic targets for cervical cancer treatment.

## 1 Introduction

Cervical cancer continues to present significant global health challenges, despite a notable decline in its incidence over the past three decades.^1^ While conventional therapeutic approaches, including surgery, radiotherapy, and chemotherapy, have demonstrated efficacy, they are frequently associated with substantial adverse effects that compromise patients’ quality of life, reduce treatment tolerance, and limit long-term survival outcomes.^2−4^ These limitations underscore the critical need for innovative, targeted therapeutic agents with improved safety profiles. Hederagenin (Hed), a natural triterpenoid, has emerged as a promising anticancer candidate due to its unique multipathway mechanism of action.^5,6^ Extensive in vitro investigations have consistently demonstrated Hed’s potent inhibitory effects across various human tumor cell lines, with in vivo studies further corroborating its robust antitumor efficacy in multiple tumor models.^7^ Notably, Hed exhibits minimal cytotoxicity in normal cell lines,^8^ and acute systemic toxicity assessments in murine models have revealed no significant toxicity or mortality,^5^ thereby establishing a favorable safety profile for potential clinical applications. Furthermore, Hed’s superior pharmacokinetic properties suggest its potential for long-term and effective tumor treatment.^9^

The mechanistic underpinnings of Hed’s antitumor activity are multifaceted. It regulates the cell cycle, induces cell arrest and inhibits tumor cell proliferation.^10^ Additionally, Hed promotes apoptotic processes through modulation of associated proteins and activation of mitochondrial pathways.^11^ The compound also demonstrates regulatory effects on autophagy, reverses chemotherapeutic resistance, and exhibits both antioxidant and anti-inflammatory properties, thereby providing comprehensive protection in antitumor treatment.^12^ Of particular interest is Hed’s potential role in modulating mitophagy, a critical process for maintaining mitochondrial homeostasis and regulating tumor cell metabolism, proliferation, and apoptosis.^13^ Recent advances in cancer biology have revealed the complex, bidirectional regulatory mechanisms of mitophagy in malignancies, including cervical cancer. While mitophagy can function as a tumor-suppressive mechanism by inhibiting cellular proliferation,^14−16^ its aberrant activation may paradoxically facilitate tumor growth and confer resistance to apoptosis.^17−19^ Moreover, Hed has demonstrated efficacy in mitigating mitochondrial dysfunction in Parkinson’s disease through the induction of mitophagy.^20^ Although oncological applications remain underexplored, it is hypothesized that Hed may modulate mitophagy by influencing the expression or functionality of mitophagy-related proteins, potentially disrupting the autophagosome-lysosome fusion process and compromising tumor cell energy metabolism and survival mechanisms.

Hypoxia, a hallmark of the tumor microenvironment, plays a critical role in cancer progression and therapeutic resistance. In cervical cancer, hypoxia stabilizes HIF-1α, which promotes angiogenesis, metabolic reprogramming, and invasion.^21^ Additionally, hypoxic conditions can induce mitophagy as an adaptive response to maintain cellular homeostasis under metabolic stress.^14,19,22^ However, dysregulated mitophagy under hypoxic conditions may also contribute to tumor cell survival and resistance to therapy.^17,23^ Therefore, understanding the interplay between hypoxia and mitophagy is crucial for developing effective therapeutic strategies targeting the hypoxic tumor microenvironment.

To comprehensively investigate Hed’s regulatory mechanism on mitophagy in the cervical cancer cell line SiHa cells, this study employs an innovative approach combining tandem mass tag (TMT) proteomics with network association algorithm (NAA) analysis. Proteomics can capture proteome changes postdrug intervention, while network analysis reveals underlying mechanisms.^24,25^ Therefore, this study aims to systematically explore Hed’s impact on mitophagy in SiHa cells. It deepens cancer understanding and supports new drug development, potentially improving treatment and quality of life for cervical cancer patients.

## 2 Materials and Methods

### 2.1 Drug Preparation

To prepare the experimental solutions, Hed powder (MedChemExpress, USA), featuring a purity of 99.95%, was first dissolved in dimethyl sulfoxide (DMSO, Sigma-Aldrich, USA) to create a concentrated stock solution of 10 mM. This stock solution was then meticulously diluted in the appropriate culture medium to achieve the desired concentrations for various experimental setups. The resulting solutions were used to study the effects of Hed under controlled conditions.

### 2.2 Cell Culture

Cervical cancer cell line SiHa cells (ATCC, USA) were cultured in Dulbecco’s Modified Eagle’s Medium (DMEM, Gibco, China) containing 10% fetal bovine serum (FBS, Gibco, USA), while human cervical immortalized squamous cell line ECT1/E6E7 cells (ATCC, USA) were cultured in Roswell Park Memorial Institute 1640 medium (RPMI 1640, Gibco, China) containing 10% FBS (Gibco, USA). The culture conditions were maintained at 37 °C with 5% CO_2_ in a humidified incubator to mimic the in vivo cellular growth environment. To ensure cell viability and experimental accuracy, cells were passaged every 3–4 days to maintain them in the logarithmic growth phase. All cellular experiments were conducted using cells at this stage to ensure reliability and reproducibility of the results.

### 2.3 Cell Viability Assay

SiHa and ECT1/E6E7 cells in logarithmic growth were seeded into 96-well plates at 5 × 10^4^ cells/mL and incubated for 24 h. The experiment was divided into blank, control, and experimental groups (each with 6 replicates). The blank group contained medium only, the control group lacked Hed, and the experimental groups were exposed to various Hed concentrations (50–150 μM). After 24 and 48 h of treatment, 10 μL of cell counting kit-8 (CCK-8, Biosharp, China) reagent was added under dark conditions, followed by a 1.5-h incubation. The absorbance (OD) at 450 nm was measured to quantify cell proliferation, and cell viability was calculated using the formula: CV (%) = [(OD_Drug_ - OD_Blank_)/(OD_Control_ - OD_Blank_)] × 100%. The half-maximal inhibitory concentration (IC_50_) was determined by fitting the dose–response curve using GraphPad Prism (Version 8.02). The entire experiment was repeated three times to ensure reliability and reproducibility of the results.

### 2.4 Annexin V-FITC/PI Fluorescence Staining

SiHa cells in logarithmic growth phase were seeded into 6-well plates at a density of 2 × 10^5^ cells/mL and cultured for 24 h. Subsequently, the experiment was stratified into a control group that received fresh medium and multiple experimental groups treated with medium containing the IC_10_, IC_50_ and IC_90_ concentration of Hed for an additional 48 h. After treatment, the cells were harvested and subjected to staining using an Annexin V-FITC/PI Apoptosis Detection Kit (Biosharp, China), adhering strictly to the manufacturer’s guidelines. Subsequently, flow cytometry analysis was conducted to classify the stained cells into four distinct quadrants based on Annexin V and PI staining patterns: Q1 (UL, Annexin V-/PI+) for dead cells or fragments, Q2 (LL, Annexin V-/PI-) for live cells, Q3 (UR, Annexin V+/PI+) for late apoptotic/necrotic cells, and Q4 (LR, Annexin V+/PI-) for early apoptotic cells. This analysis enabled quantification of the proportion of apoptotic cells induced by Hed treatment in SiHa cells.

### 2.5 TUNEL and DAPI Fluorescence Staining

Logarithmically growing SiHa and ECT1/E6E7 cells were seeded in 12-well plates at a concentration of 5× 10^4^ cells/mL. Following 24 h of routine culturing to ensure stable growth, the experiment was divided into two groups: a control group and a Hed group. The control group received fresh medium, while the Hed group was treated with medium containing the IC_50_ concentration of Hed. After 48 h of drug exposure, both groups underwent TUNEL and DAPI staining using the One-Step TUNEL/DAPI Apoptosis Assay Kit (Beyotime, China), following the manufacturer’s instructions. Subsequently, the stained cells were observed and photographed under a fluorescence microscope, revealing apoptotic cells with green fluorescence against the blue fluorescent background of all cell nuclei.

### 2.6 JC-1 Fluorescent Staining

SiHa cells in the logarithmic growth phase were seeded into 6-well plates at a concentration of 2 × 10^5^ cells/mL. After 24 h of routine culturing, the experiment was divided into four groups: a normoxic control group, a normoxic Hed group, a hypoxic control group, and a hypoxic Hed group. The hypoxic environment was simulated by adding 200 μM CoCl_2_. The control groups received fresh medium, and the Hed groups were treated with medium containing the IC_50_ concentration of Hed. After 48 h, staining was performed using the JC-1 Mitochondrial Membrane Potential Detection Kit (Biosharp, China) according to the manufacturer’s instructions. Subsequently, the stained cells were observed and photographed under a fluorescence microscope, and changes in membrane potential were analyzed using a fluorescence microplate reader and flow cytometry.

### 2.7 Mito-Tracker Green and Lyso-Tracker Red Fluorescent Staining

SiHa cells in the logarithmic growth phase were seeded into 6-well plates at a concentration of 1 × 10^5^ cells/mL. After 24 h, the experiment was divided into four groups: a normoxic control group, a normoxic Hed group, a hypoxic control group, and a hypoxic Hed group. The hypoxic environment was simulated by adding 200 μM CoCl_2_. The control groups were refreshed with fresh medium, while the Hed groups were treated with medium containing the IC_50_ concentration of Hed. After 48 h, staining was performed with Mito-Tracker Green, Lyso-Tracker Red, and Hoechst (Beyotime, China), following the manufacturer’s instructions. Upon completion of staining, the cells were observed under a fluorescence microscope with red, green, and blue filters. The mitochondria appeared green, lysosomes appeared red, and the cell nuclei appeared blue. Finally, mitochondria-lysosome colocalization analysis was performed using ImageJ software, and mitochondrial and lysosomal fluorescence intensities were measured separately for 40 randomly selected cell samples.

### 2.8 TMT-Based Quantitative Proteomics Analysis

During the experiment, SiHa cells in logarithmic growth phase were seeded uniformly at a density of 2 × 10^5^ cells/mL into 75 cm^2^ culture flasks. After 24 h of incubation, the control group was replenished with fresh medium, while the treatment group was replaced with medium containing IC_50_ concentration of Hed. After 48 h of culture, cells from both groups were collected and washed three times with ice-cold PBS. Total proteins were extracted using DB lysis buffer (8 M Urea, 100 mM TEAB, pH 8.5), followed by centrifugation at 12,000 g for 15 min at 4 °C. Protein concentration was determined using the Bradford Protein Assay Kit (Beyotime, China) according to the manufacturer’s instructions, with reference to a BSA standard curve (0–500 μg/mL).

For each group, 100 μg of protein was dissolved in 100 μL DB buffer, followed by trypsin digestion in 100 mM TEAB at 37 °C for 4 h. Additional trypsin and CaCl_2_ were added for overnight digestion. After acidification with formic acid (pH < 3), samples were centrifuged at 12,000 g for 5 min. The supernatant was desalted using a C_18_ column, washed with 0.1% formic acid/3% acetonitrile, and eluted with 0.1% formic acid/70% acetonitrile. Eluents were lyophilized, reconstituted in 0.1 M TEAB, and labeled with TMT reagent (Thermo Scientific, USA) in acetonitrile for 2 h at room temperature. The reaction was quenched with 8% ammonia, and all labeled samples were pooled, desalted, and lyophilized.

Subsequently, the labeled proteins were fractionated into 10 distinct fractions using a Rigol L3000 HPLC system equipped with a Waters BEH C_18_ column (4.6 × 250 mm, 5 μm) and a specific elution gradient. Each fraction was lyophilized, dissolved, and analyzed by LC-MS utilizing an EASY-nLC 1200UHPLC system (Thermo Fisher) coupled with a Q Exactive HF-X mass spectrometer (Thermo Fisher) in the data-dependent acquisition (DDA) mode. A 1 μg sample was injected into a homemade C_18_ Nano-Trap column (4.5 cm × 75 μm, 3 μm) and separated on a homemade analytical column (15 cm × 150 μm, 1.9 μm) with a linear gradient. Peptides were analyzed in full scan mode (*m*/*z* 350–1500, 60,000 resolution) and the top 40 precursors were selected for HCD fragmentation (30,000 resolution, 32% collision energy). Raw data were saved in “.raw” format.

The spectra were searched against the UniProt database (https://www.uniprot.org/) using Proteome Discoverer 2.4 (Thermo) with precursor ion tolerance of 10 ppm and product ion tolerance of 0.02 Da. Fixed modification was carbamidomethyl, while dynamic modifications included methionine oxidation and TMT labeling. N-terminal modifications (acetylation, TMT labeling, Met-loss, and Met-loss+acetyl) were specified. A maximum of 2 missed cleavages were allowed. PSMs with >99% confidence, proteins with ≥ 1 unique peptide, and FDR ≤ 1% were retained. Differentially expressed proteins (DEPs) were defined as those with *p* < 0.05 and |log_2_*FC*| > 0.

### 2.9 Western Blot

Logarithmically growing SiHa cells were harvested and seeded into 6-well plates at a concentration of 2 × 10^5^ cells/mL. After routine incubation for 24 h, the experiment was divided into four groups: normoxic control, normoxic Hed treatment, hypoxic control, and hypoxic Hed treatment. Hypoxic conditions were simulated by adding 200 μM of CoCl_2_. The control groups were supplied with fresh culture medium, while the Hed groups were treated with culture medium containing IC_50_ concentration of Hed. After 48 h, total protein was extracted and quantified. Subsequently, 30 μg of protein from each lane was subjected to SDS-PAGE gel electrophoresis and then transferred to a PVDF membrane. The membrane was blocked with 5% skimmed milk powder at room temperature (37 °C) for 1 h. Following this, primary antibodies against β-Actin, LC3B, p62, HIF-1α, STAT3, p-STAT3 (S727), SRC, p-SRC (Y419), AKT1, and p-AKT1 (S473) (Servicebio and Cohesion, China) were added. The dilution ratio for the internal reference β-Actin was 1:2000, and the other antibodies were diluted at 1:1000. The membrane was incubated overnight at 4 °C, followed by three washes with TBST for 10 min each. Corresponding secondary antibodies HRP conjugated Goat Anti-Mouse IgG and HRP conjugated Goat Anti-Rabbit IgG (Servicebio, China, diluted 1:5000) were added, and the membrane was incubated on a rocker at room temperature for 1 h. After three additional washes with TBST, the membrane was visualized using ECL enhanced chemiluminescence reagents. Band quantification was performed using ImageJ software.

### 2.10 Pathways Association Analysis

First, we perform KEGG pathway enrichment analysis on the differential proteins obtained from TMT proteomics using the David database (https://david.ncifcrf.gov/). This analysis screens out statistically significant pathways with *p* < 0.05. We then select individual signal transduction pathways and their combinations, conducting pairwise association analyses with cellular process pathways. Utilizing the Metascape database (https://metascape.org/), we construct individual networks for each of these pathways and their associated network, choosing the Physical Core as the data source.

**Figure 1:**
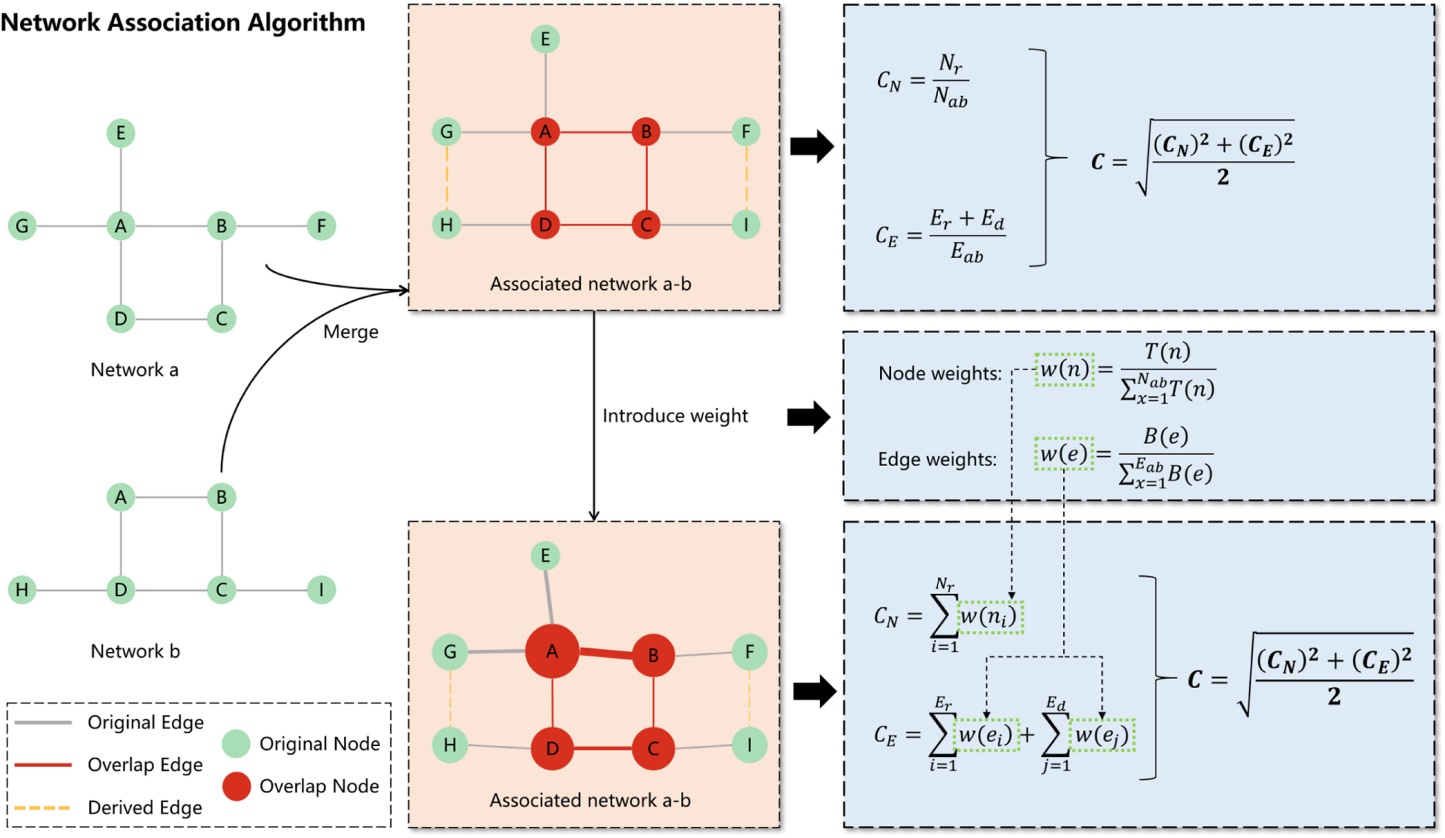
Schematic diagram of the network association algorithm (NAA).

Here, we have devised a NAA specifically designed to calculate the degree of association between different pathway combinations, as illustrated in Figure 1. Assuming that networks a and b have node counts of *N*_*a*_ and *N*_*b*_, respectively, they are reconstructed based on protein interactions to form an associated network a-b with a node count of *N*_*ab*_ and an edge count of *E*_*ab*_. Within this associated network a-b, we identify the number of overlapping nodes *N*_*r*_, the number of overlapping edges *E*_*r*_, and the number of derived edges *E*_*d*_. The degree of association between networks a and b is represented by the association coefficient *C*, which can describe the extent of the relationship between the two networks from both the node and edge perspectives.

The node association coefficient *C*_*N*_ can be expressed as the ratio of the number of overlapping nodes *N*_*r*_ to the total number of nodes *N*_*ab*_ in the associated network a-b, as shown in eq 1:
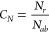
1

This formula quantifies the extent to which nodes in networks a and b overlap within the context of their associated network. A higher *C*_*N*_ value indicates a greater degree of node overlap, suggesting a stronger connection between the two networks at the node level.

The edge association coefficient *C*_*E*_ can be expressed as the ratio of the sum of the number of overlapping edges *E*_*r*_ and the number of derived edges *E*_*d*_ to the total number of edges *E*_*ab*_ in the associated network a-b, as shown in eq 2:
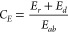
2

This formula captures the extent to which edges in networks a and b contribute to the connectivity within the associated network a-b. A higher *C*_*E*_ value indicates that a significant portion of the edges in the associated network are either overlapping or derived from the original networks, suggesting a stronger connection between the two networks at the edge level.

To summarize, the overall association coefficient *C* between networks a and b is defined as the square root of the average of the squares of the node association coefficient *C*_*N*_ and the edge association coefficient *C*_*E*_, as shown in eq 3:
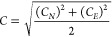
3

This combined measure provides a comprehensive assessment of the degree of association between the two networks, taking into account the contributions of both node and edge.

Indeed, in the special case where the associated network a-b has no edges (i.e., *E*_*ab*_ = 0), the edge association coefficient *C*_*E*_ becomes undefined or irrelevant since there are no edges to consider. In such a scenario, the overall association between networks a and b is primarily determined by the overlap of their nodes. Therefore, under the condition that *E*_*ab*_ = 0, the association coefficient C simplifies to the node association coefficient *C*_*N*_, as there are no edges to contribute to the overall association. So, the formula for *C* in this special case would be as shown in eq 4:
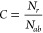
4

Here, *C* directly reflects the proportion of nodes that are shared between the two networks. In practice, however, it is unlikely that an associated network would have no edges at all, especially if the original networks a and b had significant connectivity and overlap. Nonetheless, the theoretical consideration of this special case ensures that the association coefficient *C* remains well-defined and interpretable across a range of network structures.

Furthermore, we consider the fact that different nodes and edges in the associated network may possess varying degrees of importance. To accommodate this, we introduce weights for each node and edge, which will be used to readjust the algorithm.

For the node association coefficient, we can assign a weight *w*_*n*_ to each node in the associated network. The weighted node association coefficient *C*_*Nw*_ can then be defined as the weighted sum of overlapping nodes divided by the weighted sum of all nodes in the associated network, as shown in eq 5:
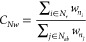
5

Similarly, for the edge association coefficient, we can assign a weight *w*_*e*_ to each edge in the associated network. The weighted edge association coefficient *C*_*Ew*_ can be defined as the weighted sum of overlapping edges and derived edges divided by the weighted sum of all edges in the associated network, as shown in eq 6:
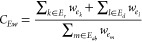
6

Finally, the overall weighted association coefficient *C*_*w*_ can be defined as the square root of the average of the squares of *C*_*Nw*_ and *C*_*Ew*_, similar to the unweighted case, as shown in eq 7:
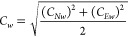
7

In this context, for a node *n*, we assign a weight based on its TOPSIS score *T(n)*, while for an edge *e*, we allocate a weight according to its network parameter, edge betweenness *B(e)*. Finally, we utilize the aforementioned association algorithm to calculate the correlation coefficient between different signal transduction pathways and cellular process pathways. The correlation coefficient ranges from 0 to 1, with a higher value indicating a stronger correlation between the two. This allows us to identify the signal transduction pathway that is most closely associated with a particular cellular process.

### 2.11 TOPSIS Analysis for Identifying Key Proteins

The most highly correlated pair of pathways is selected to construct an associated network, and a network analysis is performed to calculate the degree centrality, betweenness centrality, closeness centrality, clustering coefficient, and topological coefficient for each node in the network. Subsequently, the weight of each parameter is set using the entropy weight method. The entropy weight method first calculates the entropy value *e* of each indicator, as shown in eqs 8-9:
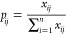
8
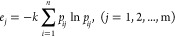
9

Then, the weight coefficient *w*of each indicator is calculated as shown in eq 10:
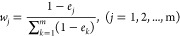
10

Next, taking these network parameters as evaluation indicators, all indicators are positively oriented and standardized. Since all the network parameters here are positive indicators, no positive orientation is required. The eq 11 of the standardization method is as follows:
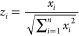
11

Afterward, the maximum value *Z*^*+*^ and minimum value *Z*^*–*^ for each indicator are determined. Then, the degree of closeness *D*^*+*^ to the maximum value and *D*^*–*^ to the minimum value for each evaluation object is determined. The eq 12 for *D*^*+*^and *D*^*–*^ are as follows:
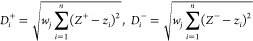
12

Next, the final evaluation index *C* for each evaluation object is calculated using the eq 13:
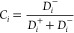
13

Finally, nodes with a final evaluation index *C* greater than 0.6 are selected as key proteins.

### 2.12 K-Core Decomposition for Determining Target Networks

An improved k-core decomposition is performed on an association network to obtain its maximum Ks-core graph. The Ks-core can be defined within a graph *G = (V, E)*, where *V = {v*_*1*_, *v*_*2*_,*···, v*_*N*_*}* represents the set of all nodes with *N* being the total number of nodes, and *E = {e*_*1*_, *e*_*2*_,*···, e*_*M*_*}* denotes the set of all edges with *M* being the total number of edges. If there exists a subgraph *G*_*k*_*= {(V*_*k*_, *E*_*k*_*), V*_*k*_*⊆ V, E*_*k*_*⊆ E}* such that for every node *v ∈ V*_*k*_, the degree of *v* is greater than or equal to *k*, then subgraph *G*_*k*_ is a Ks-core of graph *G*. Through a recursive process, all nodes and their incident edges with degrees less than *k* are removed, while preserving the key protein nodes and differential protein nodes within the network. This process continues until the largest Ks-core subgraph that cannot be further decomposed is reached, resulting in a maximum Ks-core graph where every node, excluding the key protein nodes and differential protein nodes, has a degree greater than or equal to *k*. This graph is designated as the target network. Finally, the target network is supplemented and optimized based on the biological significance of KEGG signaling pathways.

### 2.13 Molecular Docking for Predicting Potential Targets

First, the PharmMapper database (https://www.lilab-ecust.cn/pharmmapper/) was utilized to conduct virtual screening based on the structure of Hed, aiming to identify potential protein targets with a z’ score greater than 0. Subsequently, AutoDock Vina software was employed to perform high-precision molecular docking simulations between the Hed molecule and these screened protein structures.^26^ Based on the docking scoring function, potential protein targets with an affinity less than −5 kcal/mol and higher affinity compared to the original ligand were selected.

Next, these potential protein targets were further screened through two aspects. On one hand, the STRING database (https://cn.string-db.org/) was used to construct a physical network among these protein targets, followed by in-depth network analysis to uncover their associations and importance. Based on the results of the network analysis, the TOPSIS method was applied to evaluate and determine the key node proteins within the network, which play crucial roles in regulatory networks. On the other hand, information from multiple authoritative databases, including DisGeNET (https://disgenet.com/), OMIM (https://omim.org/), KEGG (https://www.genome.jp/kegg/), TTD (https://db.idrblab.net/ttd/), and MalaCards (https://www.malacards.org/), was integrated to comprehensively collect gene data related to cervical cancer. David database was then used to perform KEGG pathway enrichment analysis on these genes, selecting all proteins significantly enriched in the pathway pair with the strongest correlation. At the same time, these enriched proteins were intersected with the aforementioned potential protein targets, and the resulting overlap was considered as the predicted potential therapeutic targets for cervical cancer. Finally, the key predicted targets were determined by comprehensively considering both aspects.

### 2.14 Statistical Analysis

All data and results have been validated by at least three independent experiments. The data are presented as mean ± standard deviation (SD) and analyzed statistically using GraphPad Prism 8.0 software. The significance of statistical differences among multiple groups is assessed by one-way analysis of variance (ANOVA). A *P*-value less than 0.05 is considered statistically significant and is indicated by asterisks (^*^p < 0.05, ^**^p < 0.005, ^***^p < 0.0005, ^****^p < 0.0001).

## 3 Results

### 3.1 Hed Reduces SiHa Cell Viability and Induces Apoptosis

**Figure 2:**
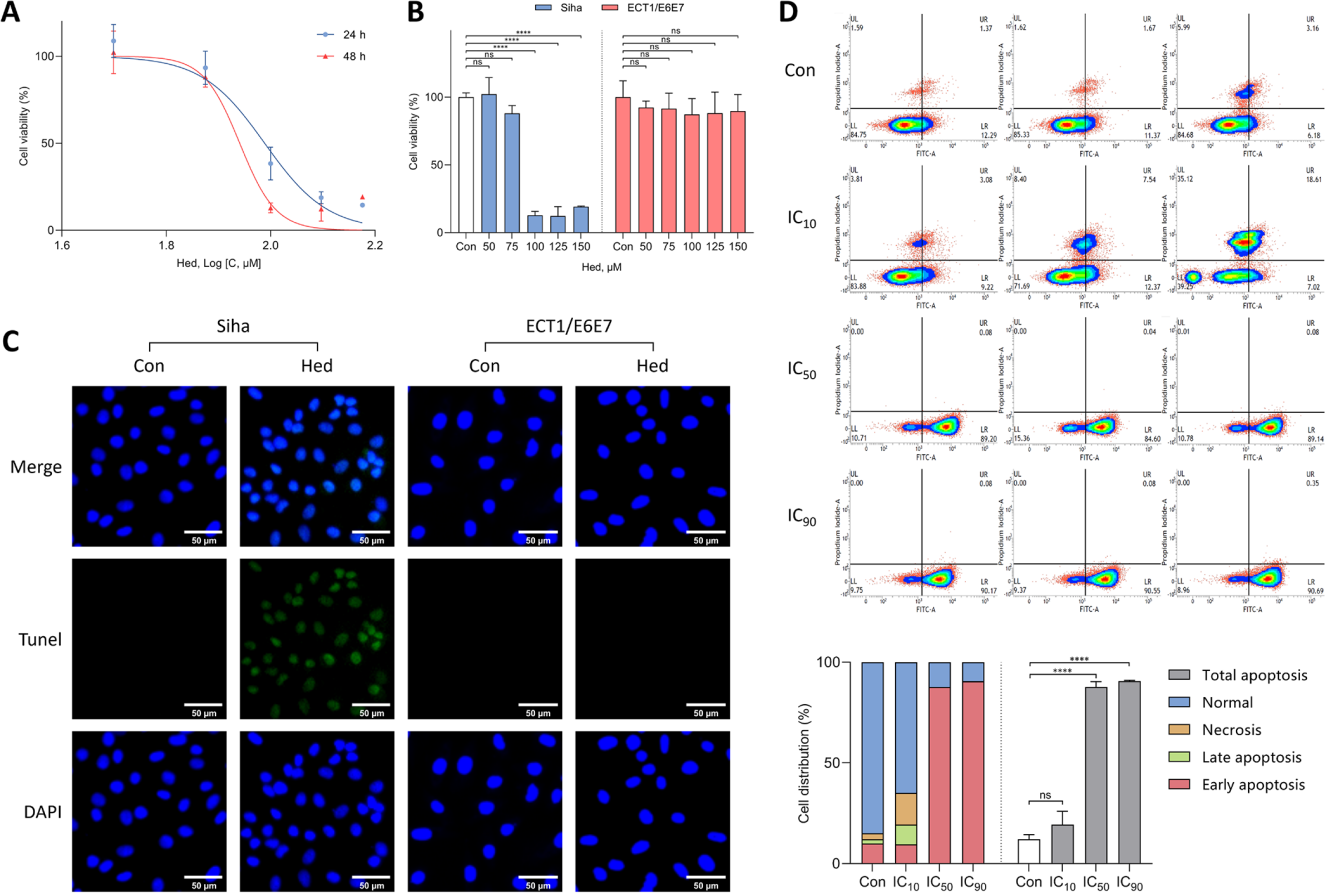
Effect of Hed on SiHa and ECT1/E6E7 cells. (A) IC_50_ fitting curves for SiHa cell viability at 24 and 48 h; (B) cell viability of SiHa and ECT1/E6E7 at concentrations of 50–150 μM; (C) SiHa and ECT1/E6E7 for TUNEL/DAPI staining; (D) SiHa cell apoptosis distribution detected by flow cytometry and analysis. Asterisks (*) indicate statistical significance with ****p < 0.0001.

We first evaluated the effect of Hed on SiHa cell viability using the CCK-8 assay. Both 24-h and 48-h treatments resulted in a dose-dependent decrease in cell survival rates. The half-maximal inhibitory concentrations (IC_50_) were calculated as 97.39 μM at 24 h and 87.24 μM at 48 h (Figure 2A). Notably, Hed concentrations ≥ 100 μM significantly suppressed SiHa cell proliferation compared to the untreated control group (*p* < 0.05), whereas no significant inhibitory effect was observed on ECT1/E6E7 cells at concentrations ranging from 50 to 150 μM (*p* > 0.05) (Figure 2B).

Consistent with these findings, TUNEL/DAPI staining revealed a marked increase in green fluorescence (indicating apoptosis) in Hed-treated SiHa cells, while ECT1/E6E7 cells showed no observable changes (Figure 2C). Further analysis by flow cytometry demonstrated a concentration-dependent shift in SiHa cell distribution to the fourth quadrant, reflecting early apoptotic events (Figure 2D). Total apoptosis rates under IC_50_ and IC_90_ treatments were significantly elevated compared to the control group, with statistically significant differences (*p* < 0.05).

### 3.2 Hed Induces Changes in Mitochondrial Membrane Potential of SiHa Cells

**Figure 3:**
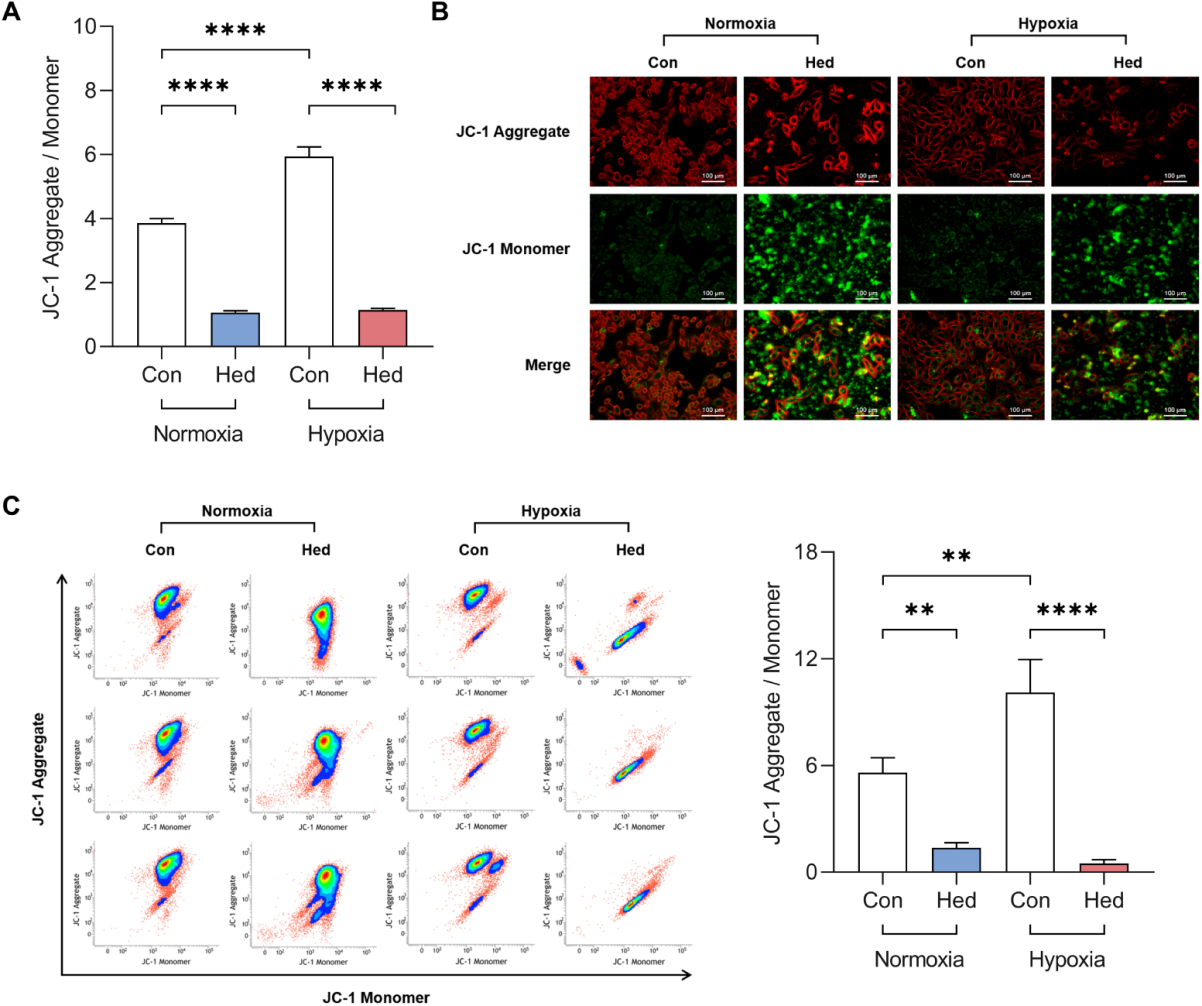
Effect of Hed on mitochondrial membrane potential in SiHa cells. (A) Results from fluorescence microplate reader; (B) results from fluorescence microscopy; (C) results from flow cytometry. Asterisks (^*^) indicate statistical significance with ^**^p < 0.005 and ^****^p < 0.0001.

To systematically investigate the effect of Hed intervention on mitochondrial membrane potential in cervical cancer SiHa cells, we employed a comprehensive approach utilizing fluorescence microplate reader, fluorescence microscopy, and flow cytometry. As shown in Figure 3A, under normoxic culture conditions, Hed intervention significantly reduced the expression level of mitochondrial membrane potential-related proteins (measured by the ratio of aggregates to monomers) in SiHa cells compared to the control group, with statistical significance (*p* < 0.05). This finding indicates that Hed can effectively modulate the stability of mitochondrial membrane potential in normoxic environments. Notably, under hypoxic culture conditions, while the ratio of aggregates to monomers in the control group increased compared to normoxia, reflecting a potential adaptive change in mitochondrial membrane potential induced by hypoxic stress, the Hed-treated group still exhibited a significant decrease, with statistical significance (*p* < 0.05). This further confirms the regulatory role of Hed on mitochondrial membrane potential under adverse conditions such as hypoxia. As depicted in Figure 3B, fluorescence microscopy directly demonstrated the effect of Hed intervention. In the Hed-treated group, the red fluorescence representing high membrane potential significantly decreased, while the green fluorescence representing low membrane potential markedly increased. This change is consistent with the quantitative results from the fluorescence microplate reader, further confirming the downregulation of mitochondrial membrane potential by Hed. The flow cytometry results (Figure 3C) concurred with the conclusions drawn from the above two methods. Regardless of normoxic or hypoxic conditions, Hed intervention led to a significant decrease in the ratio of aggregates to monomers of mitochondrial membrane potential-related proteins in SiHa cells, with statistical significance. This result not only validates the accuracy of the fluorescence microplate reader and microscopy observations but also emphasizes the stability and consistency of Hed’s regulation of mitochondrial membrane potential across different oxygen environments. In summary, this study, through multiple technical approaches, confirms that Hed can significantly reduce mitochondrial membrane potential in cervical cancer SiHa cells under both normoxic and hypoxic conditions.

### 3.3 The Effect of Hed on Mitophagy in SiHa Cells

**Figure 4:**
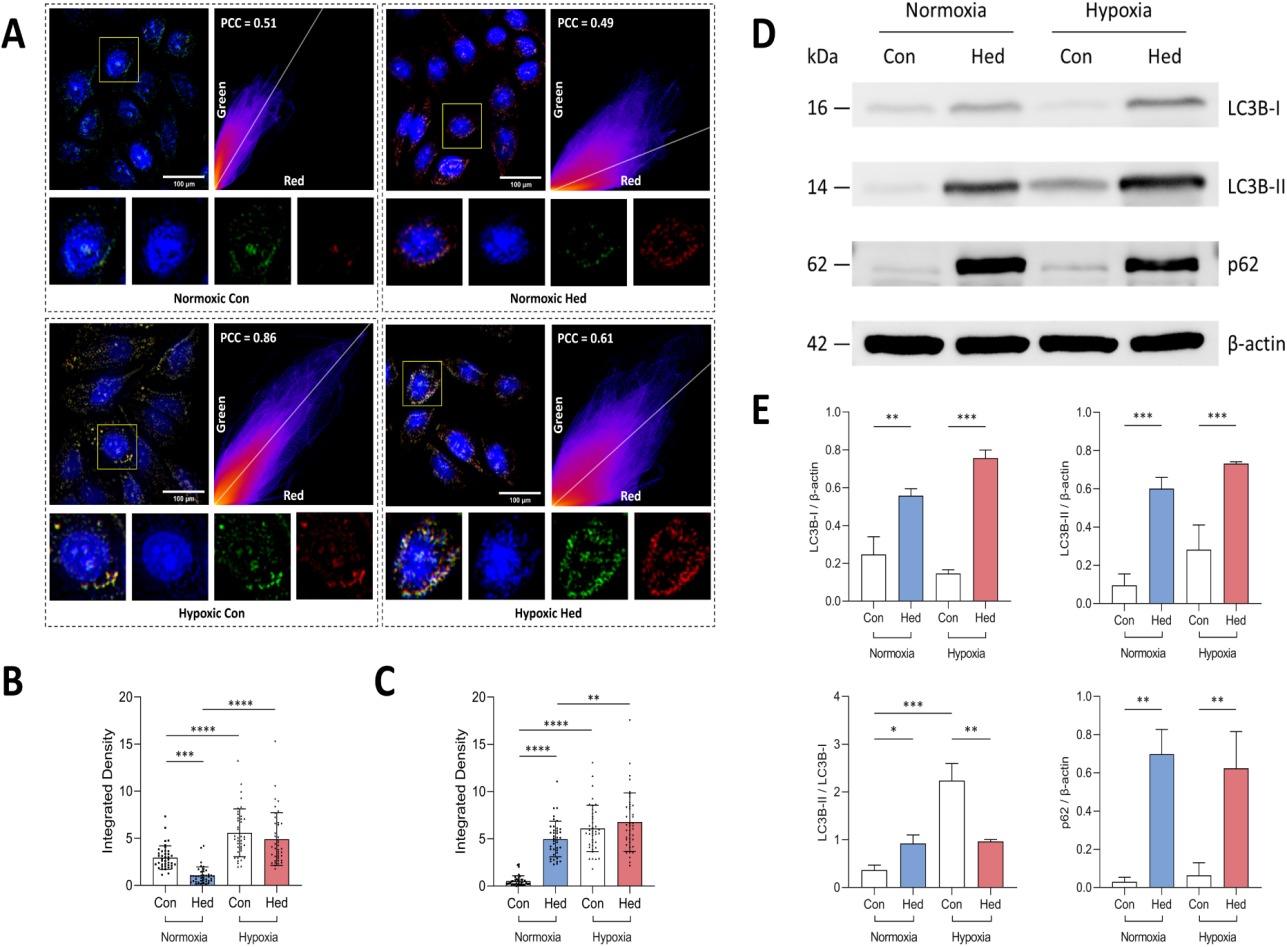
Effects of Hed on mitophagy in SiHa cells. (A) Fluorescence microscopy images showing mitochondrial (Mito-Tracker Green) and lysosomal (Lyso-Tracker Red) labeling, and PCC analysis; (B) quantitative analysis of mitochondrial fluorescence intensity; (C) quantitative analysis of lysosomal fluorescence intensity; (D) Western blot analysis of mitophagy markers LC3B and p62; (E) densitometric quantification of protein expression levels relative to β-actin. Asterisks (*) indicate statistical significance with *p < 0.05, **p < 0.005, ***p < 0.0005 and ****p < 0.0001.

To investigate Hed’s specific regulatory role in mitophagy, we systematically evaluated mitochondrial dynamics through multiple approaches. First, fluorescence colocalization analysis using Mito-Tracker Green and Lyso-Tracker Red revealed distinct mitochondrial-lysosomal interaction patterns (Figure 4A-C). Under normoxia, low Pearson colocalization coefficient (PCC, ∼ 0.5) persisted regardless of Hed treatment, suggesting minimal organelle colocalization. However, Hed induced significant mitochondrial fluorescence attenuation (*p* < 0.0005) accompanied by lysosomal signal amplification (*p* < 0.0001), with a visible color shift in the Pearson colocalization image from green to red. These observations indicate lysosomal accumulation and potential impairment of degradation processes.

Hypoxic exposure markedly enhanced mitochondrial-lysosomal interactions, evidenced by elevated PCC (0.86 in hypoxia control vs 0.51 in normoxia controls) and intensified dual-channel fluorescence. Notably, Hed intervention under hypoxia significantly suppressed this colocalization (0.61 in hypoxia Hed vs 0.86 in hypoxia control), demonstrating its inhibitory effect on hypoxia-induced mitophagy initiation.

Western blot analysis of mitophagy markers further corroborated these findings (Figure 4D-E). Normoxic Hed treatment increased LC3B–II/I conversion (*p* < 0.05) while elevating p62 accumulation (*p* < 0.005), suggesting concurrent autophagosome formation promotion and autolysosomal degradation. Hypoxia-enhanced mitophagic flux (evidenced by LC3B–II/I elevation) was significantly reversed by Hed (*p* < 0.005), accompanied by upregulated total LC3B and p62 levels, reinforcing Hed’s inhibitory effect on autophagosome-lysosome fusion under hypoxic stress.

Collectively, both colocalization patterns and molecular marker dynamics confirm Hed’s dual regulatory effects: under normoxia, it promotes autophagosome biogenesis while impairing degradation efficiency; under hypoxia, it exerts comprehensive suppression of mitophagic flux by inhibiting autophagosome-lysosome convergence. These differential responses highlight Hed’s context-dependent modulation of mitochondrial quality control mechanisms.

### 3.4 DEPs and Enriched Pathways after Hed Intervention

**Figure 5:**
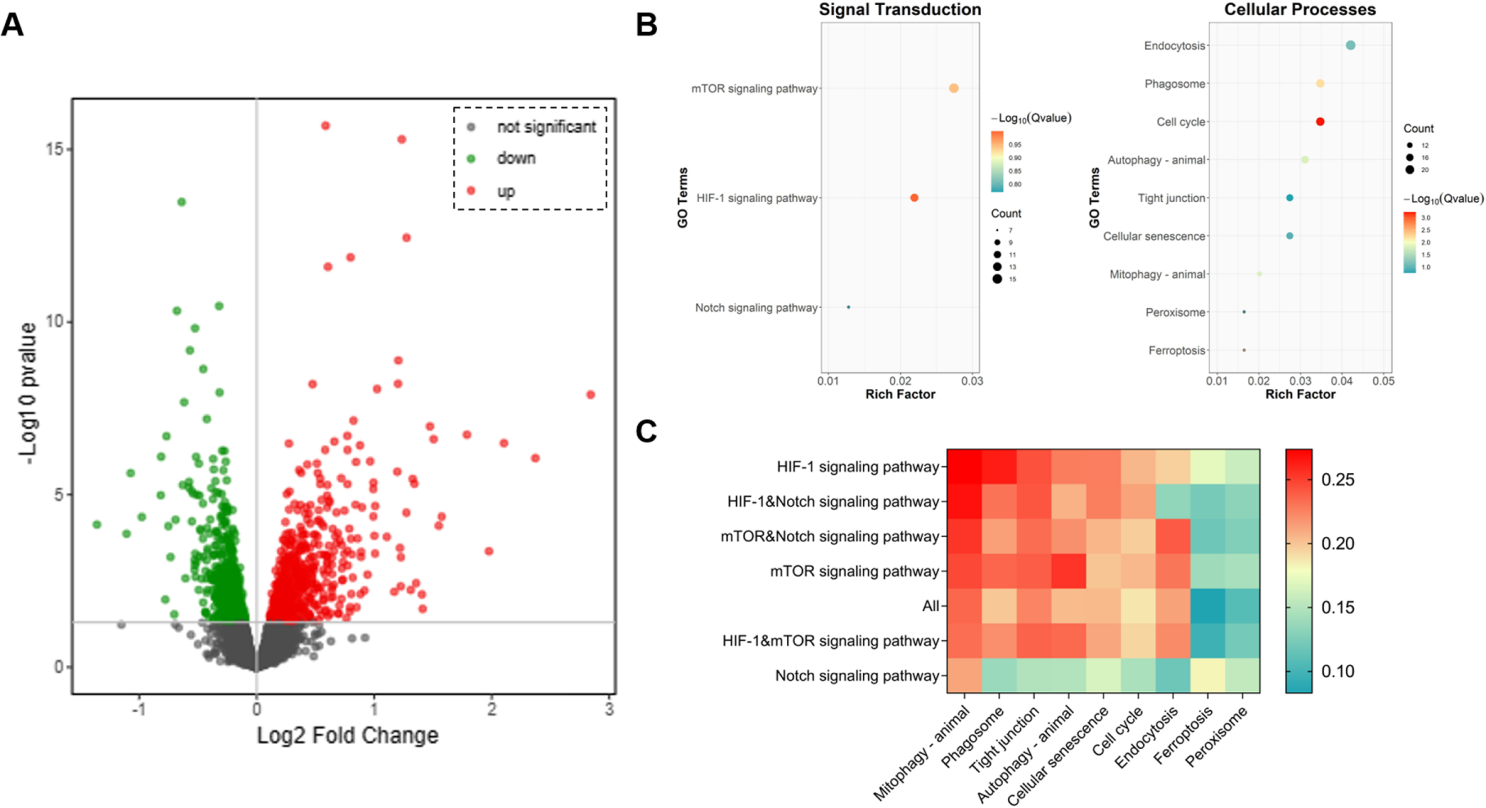
Proteomic profiling and pathway enrichment analysis following Hed treatment. (A) Volcano plot displaying DEPs; (B) signal transduction pathways and cellular processes enriched with DEPs; (C) heatmap of pathways association analysis.

Using TMT proteomics technology, we systematically analyzed Hed-treated samples and identified 6,951 unique proteins (Table S1). To investigate the regulatory mechanisms of Hed intervention on biological systems, DEPs were screened (Table S2). The volcano plot (Figure 5A) revealed 1,270 significant DEPs (655 upregulated and 615 downregulated), indicating extensive protein-level alterations induced by Hed intervention. KEGG pathway enrichment analysis of these DEPs identified 67 significantly enriched pathways (Table S3) spanning diverse biological functions. Notably, three signal transduction pathways and nine cellular process pathways (Figure 5B) were identified as potential mediators of Hed’s biological effects. Pairwise associations between the two pathway categories were quantified through pathway association analysis, with detailed results documented in Table S4. To elucidate their interrelationships, we further generated an interaction heatmap (Figure 5C) that systematically visualizes the cross-talk patterns between these distinct biological pathways. A prominent association was observed between the HIF-1 signaling pathway and mitophagy.

### 3.5 Target Network for Hed Intervention

**Figure 6:**
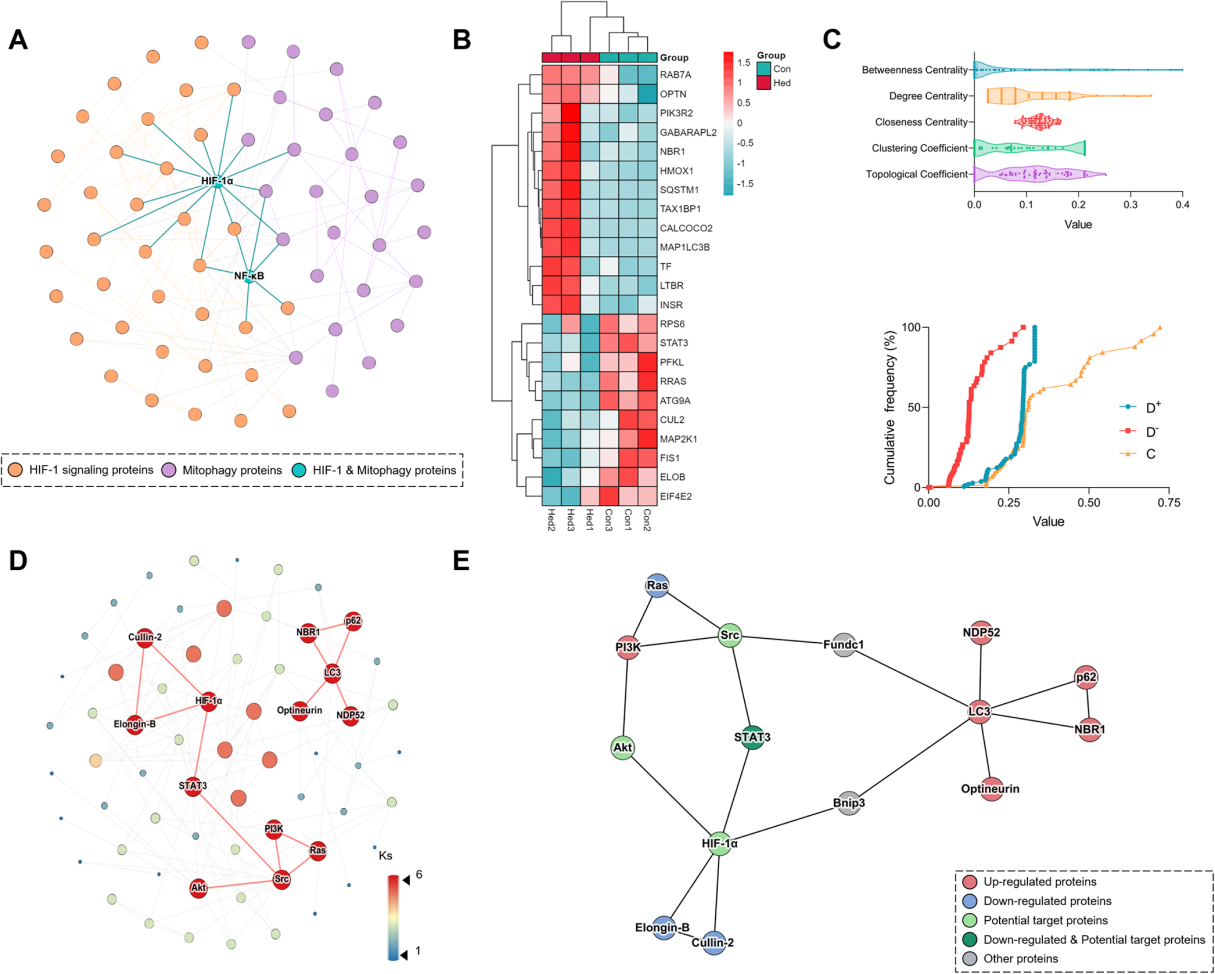
Analysis of the associative network.(A) Associative network between HIF-1 signaling pathway and mitophagy; (B) grouped clustering analysis of DEPs in associative network; (C) distribution of network parameters and cumulative frequency plot of TOPSIS scores; (D) K-core decomposition of associated network; (E) target network of Hed intervention.

As depicted in Figure 6A, an in-depth investigation of the crosstalk between the HIF-1 signaling pathway and mitophagy enabled the construction of an associative network. This network contains 64 nodes connected by 125 edges, including 38 nodes from the HIF-1 signaling pathway and 28 from mitophagy, with HIF-1α and NF-κB acting as critical bridges between these pathways. Grouped clustering analysis of DEPs within the network revealed distinct expression patterns: 13 proteins were significantly upregulated and 10 were downregulated (Figure 6B). To identify optimal intervention targets, a TOPSIS analysis prioritized SRC, STAT3, AKT1, and HIF1A as the four key targets from the network nodes (Table S5). The broad distribution of five critical network parameters and the cumulative frequency plot of TOPSIS scores further validated the discriminative power and centrality of these targets (Figure 6C). To deepen our understanding and confirm the target network structure, an enhanced k-core decomposition method uncovered a core subnetwork with a maximum coreness (Ks = 6), which consists of two tightly interconnected clusters (Figure 6D). Finally, integration of complementary bioinformatics data sets yielded a refined target network (15 nodes, 19 edges), definitively establishing SRC, STAT3, AKT1, and HIF-1α as key regulators of mitophagy in SiHa cells (Figure 6E).

### 3.6 Predicted Targets of Hed

**Figure 7:**
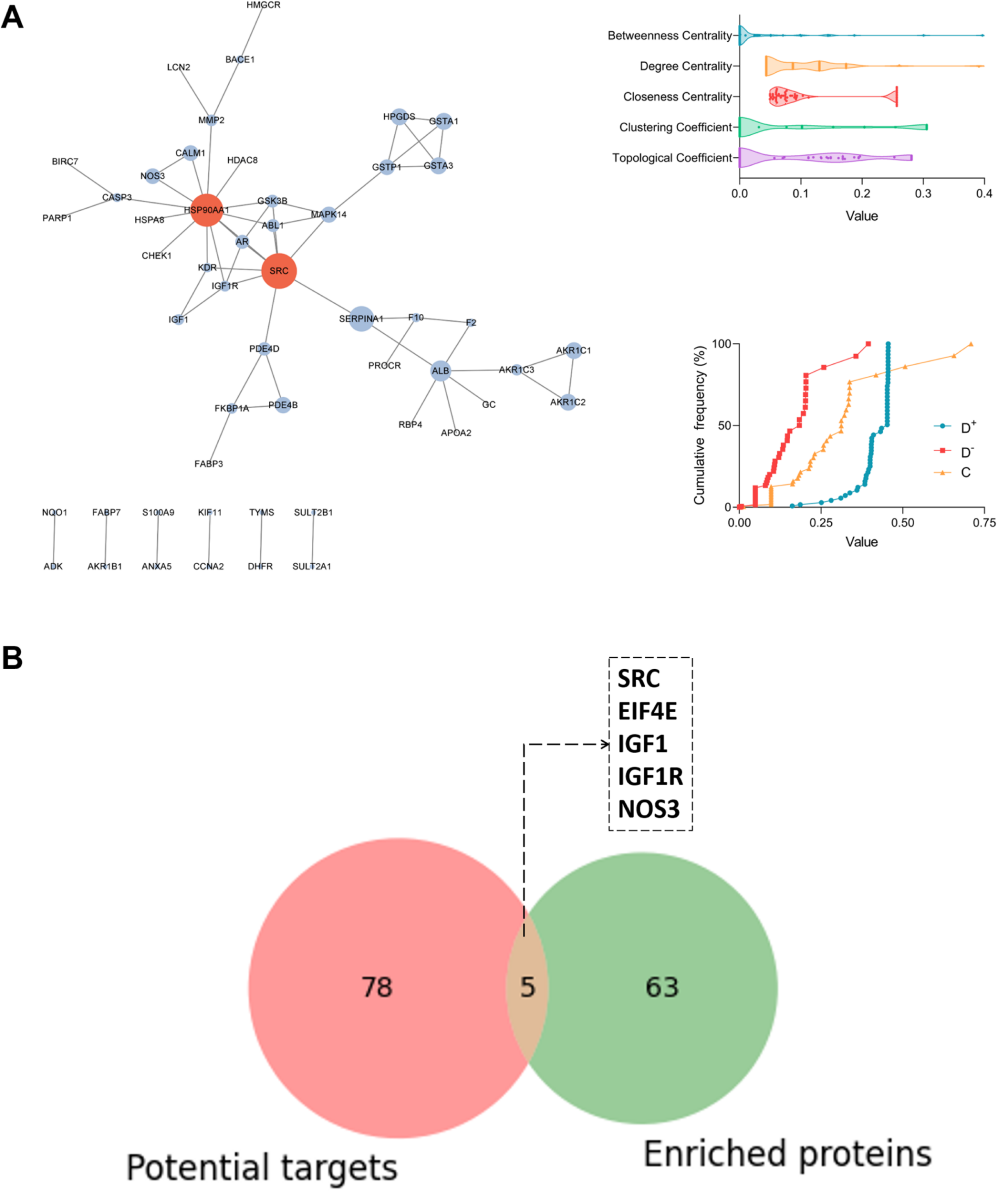
Demonstration of empirical results for Hed target prediction strategies. (A) Analysis outcomes of key nodes based on network structure; (B) interpretation of target Venn diagram based on bioinformatics.

Furthermore, based on the structural characteristics of Hed, we initially screened out 161 potential protein targets with positive z’ score values and narrowed this down to 83 targets using high-precision molecular docking techniques, and the results are presented in Tables S6–8. Subsequently, we adopted a dual screening strategy: First, we applied TOPSIS analysis based on the physical network to identify two key node proteins, SRC and HSP90AA1, from among 52 network nodes (as shown in Figure 7A and Table S9). Second, by integrating authoritative database resources, we compiled a list of 1879 cervical cancer-related proteins (displayed in Table S10), of which 68 were enriched in the associated network. After intersecting these with the 83 potential targets, we predicted five targets closely related to cervical cancer: SRC, IGF1, NOS3, EIF4E, and IGF1R (as shown in Figure 7B and Table S11). From these two different screening results, it is evident that SRC not only occupies a central position in the network but is also highly correlated with cervical cancer.

### 3.7 Key Targets for Hed Intervention

**Figure 8:**
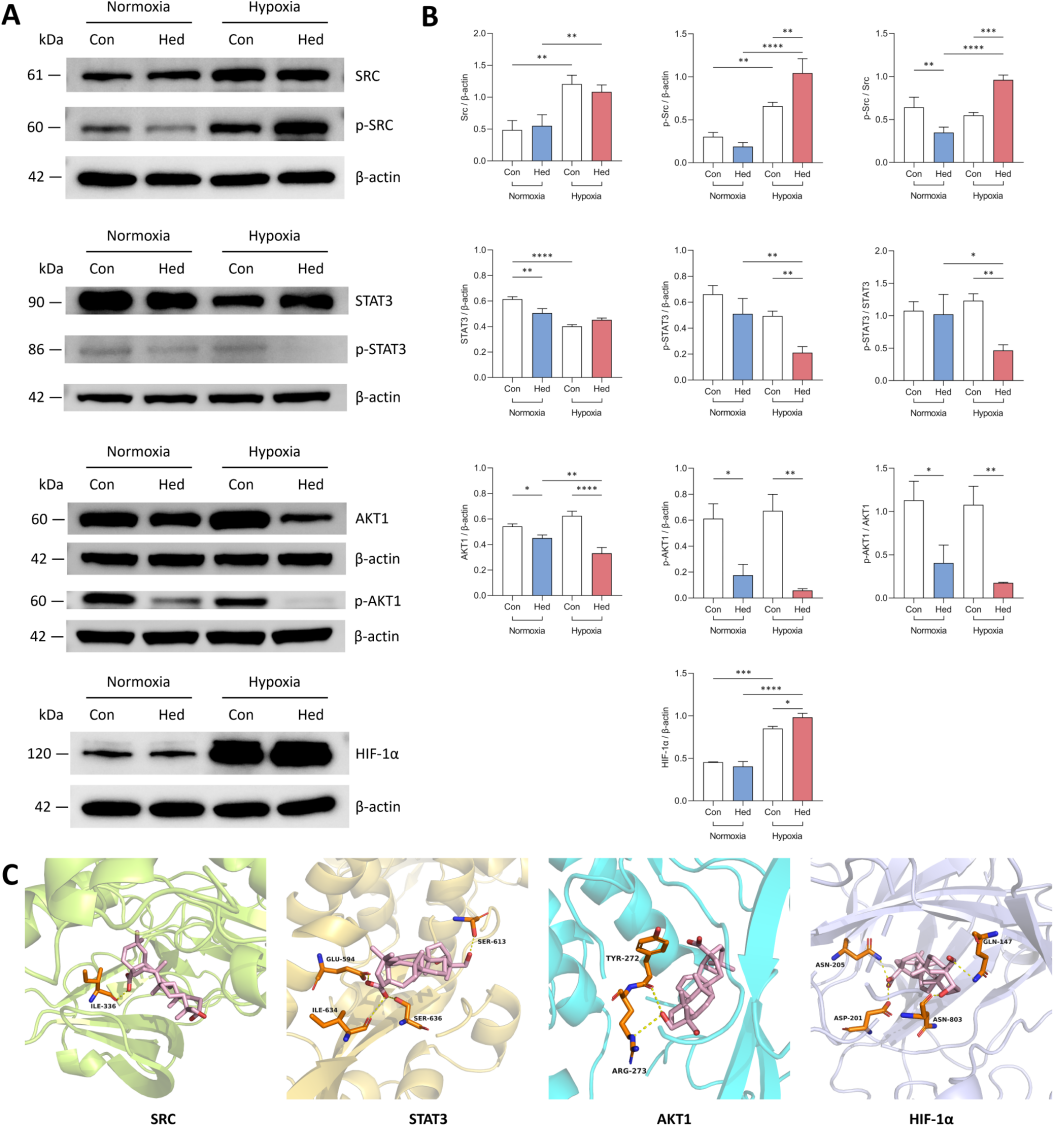
Effects of Hed intervention on key target expression and their binding modes. (A) Western blot analysis of key targets under different oxygen conditions; (B) Statistical analysis of key target expression levels. Asterisks (*) indicate statistical significance with ^*^p < 0.05, ^**^p < 0.005, ^***^p < 0.0005 and ^****^p < 0.0001; (C) Simulation of binding modes between Hed and key targets.

To thoroughly investigate the key targets of Hed’s intervention in mitochondrial autophagy, we conducted Western blot experiments to systematically assess the expression dynamics of key targets SRC, STAT3, AKT1, HIF-1α, and their phosphorylated forms before and after Hed treatment, under both normoxic and hypoxic conditions. Figure 8A,B visually present the experimental results: Under normoxic conditions, Hed significantly downregulated the expression levels of STAT3, AKT1, and its phosphorylated form p-AKT1, and reduced the ratios of p-SRC/SRC and p-AKT1/AKT1. In contrast, under hypoxic conditions, Hed promoted the expression of p-SRC and HIF-1α, increased the ratio of p-SRC/SRC, and inhibited the expression of p-STAT3, p-AKT1, and their corresponding phosphorylation ratios. Furthermore, compared to normoxic conditions, hypoxic conditions significantly upregulated the expression of SRC, p-SRC, and HIF-1α, while downregulating the expression of STAT3.

Additionally, Figure 8C provides a detailed illustration of the molecular docking between Hed and the four key targets SRC, STAT3, AKT1, and HIF-1α. Specifically, Hed binds to the ILE-336 residue of SRC through a single hydrogen bond, connects to the GLU-594, ILE-634, SER-613, and SER-636 residues of STAT3 through four hydrogen bonds, interacts with the ARG-273 and TYR-272 residues of AKT1 through two hydrogen bonds, and forms interactions with the ASN-205, ASN-803, ASP-201, and GLN-147 residues of HIF-1α through four hydrogen bonds.

## 4 Discussion

This study systematically integrates TMT-based proteomics with network pharmacology to elucidate the molecular mechanisms by which Hed regulates cervical cancer SiHa cells. Through TMT proteomic profiling, we identified 1,270 differentially expressed proteins in Hed-treated cells, providing a comprehensive landscape of proteomic alterations. However, traditional approaches based on bioinformatics annotation and pathway enrichment analysis may not be sufficient to fully elucidate the complex mechanisms of action of cellular pathway crosstalk. Meanwhile, current methods for pathway crosstalk analysis exhibit limitations. For instance, node/edge-overlap-based approaches like the Jaccard index measure pathway similarity solely through shared components, essentially performing structural comparisons while ignoring interactive relationships between pathways, and the Jaccard’s index varies strongly depending on the size of the data set.^27^ Similarly, single-metric topological evaluations on association networks (e.g., betweenness centrality analysis) fail to quantify cross-pathway associations despite network construction efforts.^28^ To address these gaps, we present NAA, which innovatively integrates multidimensional topological features (including node/edge overlaps and edge-derived relationships) with biological weights. This multidimensional framework enables quantitative characterization of pathway–pathway associations, overcoming the inherent constraints of conventional network-based methods.

Drugs generally exert top-down effects on cellular systems, initially targeting specific signaling pathways to modulate downstream cellular processes before manifesting their biological effects.^29 −31^ To systematically investigate how Hed regulates biological processes in SiHa cells through pathway interactions, we developed the NAA following rigorous pathway enrichment analysis. This innovative framework introduces two quantitative metrics: the node association coefficient *C*_*N*_ and edge association coefficient *C*_*E*_, which collectively quantify the degree of correlation between different pathway combinations. To address the inherent topological heterogeneity of biological networks, we incorporated weighted centrality metrics: topological importance T(n) for nodes and edge betweenness centrality B(e) for edges. The T(n) metric integrates five critical network parameters via the TOPSIS method—degree centrality, betweenness centrality, closeness centrality, clustering coefficient, and topological coefficient—providing a multidimensional evaluation of a node’s global influence and local connectivity. For edges, B(e) quantifies their role as critical bridges between network modules, as edge betweenness centrality has been shown to effectively identify critical pathway interactions in biological networks. This weighting scheme aligns with complex network theory, where high-T(n) nodes maintain network integrity and high-B(e) edges represent privileged communication channels.^32^ By embedding these biologically informed topological features into our algorithm, we achieved a mechanistically relevant assessment of pathway–pathway associations. The NAA enables precise calculation of association coefficients between signaling pathways and cellular process pathways, facilitating identification of key regulatory pathways governing specific biological functions. Such insights advance our understanding of disease pathogenesis, therapeutic target identification, and rational drug development by elucidating how upstream signaling events propagate through network architectures to drive cellular outcomes.

Application of NAA revealed the HIF-1 signaling pathway as the most strongly associated with mitophagy. Subsequent TOPSIS-guided screening identified four key targets (SRC, STAT3, AKT1, HIF1A) within a 15-node/19-edge target network. Target network construction incorporated a multidimensional evaluation framework where five topological parameters were objectively weighted using the entropy weight method.^33^ This data-driven approach eliminates subjective bias in parameter prioritization while ensuring comprehensive assessment of node significance.^34^ Subsequent k-core decomposition systematically pruned low-degree nodes, optimizing network topology to emphasize functionally critical elements while preserving both key regulatory proteins and differentially expressed nodes.^35^ The final architecture retains essential biological information while enhancing interpretability through structural simplification. For predictive identification of Hed’s potential targets, we employed dual orthogonal strategies: one was key node prediction based on the network structure, and the other was bioinformatics prediction based on database resources. The results of both strategies indicated that SRC occupies a core position in the network and is closely related to cervical cancer, which is highly consistent with the key targets of the target network we previously identified.

Mitophagy is a crucial process for cells to maintain mitochondrial health and function, preventing cell damage and disease occurrence by eliminating damaged or excess mitochondria.^13^ As a compound with potential anticancer activity, Hed’s impact on mitophagy is critical for understanding its anticancer mechanism. Our research has demonstrated that Hed significantly reduces the mitochondrial membrane potential of SiHa cells, a key signal that triggers mitophagy but is also closely associated with mitochondrial-mediated apoptosis.^36^ Indeed, our results revealed that Hed is involved in both mitophagy and apoptosis processes, highlighting its multifaceted role in cellular regulation.

Under normoxic conditions, Hed induced significant mitochondrial fluorescence attenuation and lysosomal signal amplification without altering the PCC, suggesting that lysosomal accumulation rather than enhanced organelle interaction dominated the observed phenotype. This paradoxical dissociation, characterized by elevated autophagosome formation (increased LC3B–II/I conversion) alongside impaired autophagolysosome degradation (p62 accumulation), reveals a bifurcated regulatory mechanism. Hed appears to concurrently stimulate autophagosome biogenesis while disrupting lysosomal clearance under basal conditions, resulting in stalled mitophagic flux. The visible color shift in PCC imaging from green (mitochondrial dominance) to red (lysosomal dominance) further supports lysosomal overload rather than functional mitophagy progression.

In hypoxic contexts, where mitochondrial-lysosomal interactions were intrinsically amplified, Hed exerted a striking inhibitory effect, suppressing both colocalization and hypoxia-driven LC3B–II/I elevation. This dual suppression, targeting both autophagosome-lysosome convergence and mitophagic flux, contrasts sharply with its normoxic actions. While hypoxia typically enhances mitochondrial quality control via accelerated autophagic turnover,^37^ Hed disrupted this adaptive response. The observed upregulation of HIF-1α by Hed under hypoxia suggests a potential crosstalk with oxygen-sensing pathways, possibly redirecting cellular priorities away from mitophagy toward alternative survival strategies.

Further analysis revealed that Hed modulates mitophagy through interconnected pathways, with the HIF-1 signaling pathway acting as a central role. Under normoxic conditions, Hed significantly suppressed the expression of STAT3, AKT1, and its phosphorylated form p-AKT1, suggesting its inhibitory effect on these signaling proteins. Notably, Hed also reduced the p-SRC/SRC and p-AKT1/AKT1 phosphorylation ratios, indicating dual regulation of both total protein levels and activation states. Given the critical roles of SRC, STAT3, and AKT1 in cellular proliferation, survival, and migration,^38 −40^ these findings imply that Hed disrupts normoxic cellular homeostasis by targeting these pathways, thereby linking their inhibition to mitophagy regulation. Under hypoxic conditions, Hed exhibited distinct regulatory effects. While it enhanced the expression of p-SRC and HIF-1α and elevated the p-SRC/SRC ratio, a hallmark of hypoxia adaptation, it maintained its inhibitory effects on p-STAT3 and p-AKT1 phosphorylation. The upregulation of HIF-1α, a master regulator of hypoxic responses,^41^ suggests that Hed potentiates cellular adaptation to hypoxia through HIF-1α activation. Conversely, the sustained suppression of STAT3 and AKT1 signaling highlights Hed’s persistent interference with these pathways regardless of oxygen tension, reinforcing their mechanistic connection to mitophagy regulation.

Molecular docking studies further elucidated Hed’s mode of action. Hed formed hydrogen bonds with key residues of SRC, STAT3, AKT1, and HIF-1α, likely altering their conformational dynamics and functional states. Strikingly, multiple hydrogen bonds were observed between Hed and HIF-1α, providing a structural basis for its hypoxia-specific upregulation of HIF-1α. Similarly, interactions with SRC, STAT3, and AKT1 may directly impair their phosphorylation capacity, aligning with the observed reduction in activated protein levels. These findings position Hed as a multitarget modulator capable of fine-tuning both hypoxic adaptation and mitophagy through pathway-specific interactions.

However, this study also has certain limitations. First, this study was mainly conducted at the cellular level, and further validation of Hed’s antitumor effects in animal models or clinical trials is needed. Meanwhile, our research focused on cervical cancer SiHa cells, and Hed’s regulatory role in mitophagy may differ in other types of cancer cells or normal cells. Therefore, future research can further explore Hed’s regulatory role in mitophagy in other cell types or animal models. Second, our NAA may be limited in practical applications. For example, the weight assignment of nodes and edges in the correlation network may be influenced by various factors, including data completeness, differences in experimental conditions, and biological individual differences. Therefore, caution should be exercised during the weight assignment process to ensure the accuracy and reliability of the results.

## 5 Conclusion

In summary, this study has unveiled the potential mechanism by which Hed regulates mitophagy, demonstrating that it modulates the homeostasis of the entire target network by influencing the expression and phosphorylation states of key targets, thereby profoundly affecting the process of mitophagy. These findings not only provide a new perspective for understanding the anticancer mechanisms of Hed but also offer significant clues for further research and development of therapeutic strategies targeting mitophagy-related diseases. Future studies can further explore the regulatory role of Hed in mitophagy in other cell types or disease models, as well as its interactions with other signaling pathways, offering new perspectives and methodologies for a deeper understanding of the regulatory mechanisms of mitophagy and the treatment of related diseases.

## Data Availability

The mass spectrometry proteomics data have been deposited to the ProteomeXchange Consortium (https://proteomecentral.proteomexchange.org) via the iProX partner repository^42,43^ with the data set identifier PXD059535.

## Supporting Information Available

Information on identified proteins (Table S1) (XLSX). Information on identified differential proteins (Table S2) (XLSX). Results of KEGG enrichment analysis (Table S3) (XLSX). Results of pathways association analysis (Table S4) (XLSX). Results of TOPSIS analysis of associative network (Table S5) (XLSX). Results of PharmMapper (Table S6) (XLSX). Results of molecular docking (Table S7) (XLSX). Results of screening (Table S8) (XLSX). Results of TOPSIS analysis of physical network (Table S9) (XLSX). Information on cervical cancer-related proteins (Table S10) (XLSX). Results of intersecting protein (Table S11) (XLSX). Original Western blot (Figures S1–S3) (ZIP)
