# Supplementary material for: Elucidating the Molecular Mechanisms of Hederagenin-Regulated Mitophagy in Cervical Cancer SiHa Cells through an Integrative Approach Combining Proteomics and Advanced Network Association Algorithm
Source: J Proteome Res. 2025 Mar 26;24(4):2081–95. doi: 10.1021/acs.jproteome.5c00022 (PMC11976847; doi:10.1021/acs.jproteome.5c00022)

Figure S1. Original Western blot of LC3B-I, LC3B-II, p62, p-AKT.


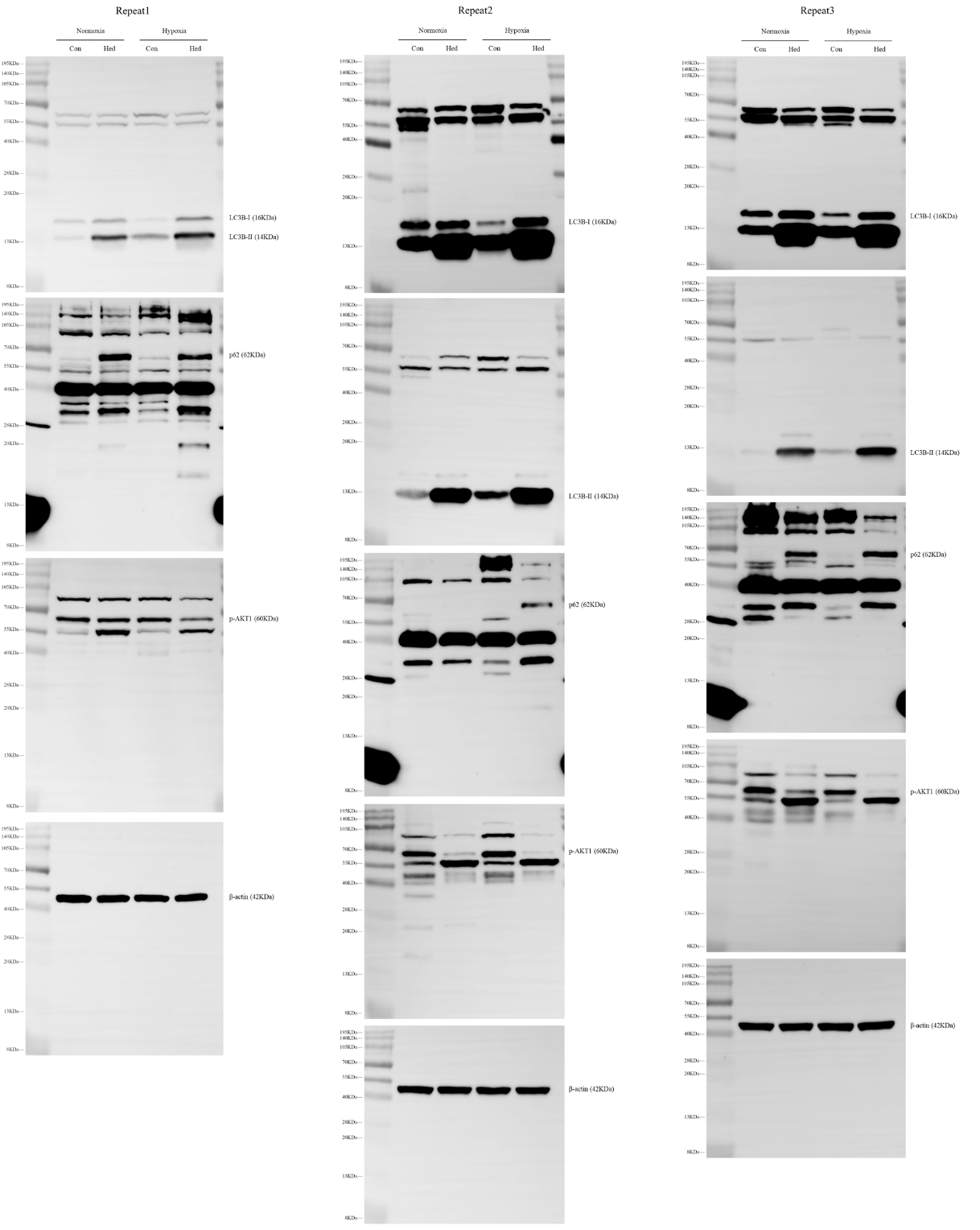


Figure S2. Original Western blot of SRC, p-SRC, HIF-1α.


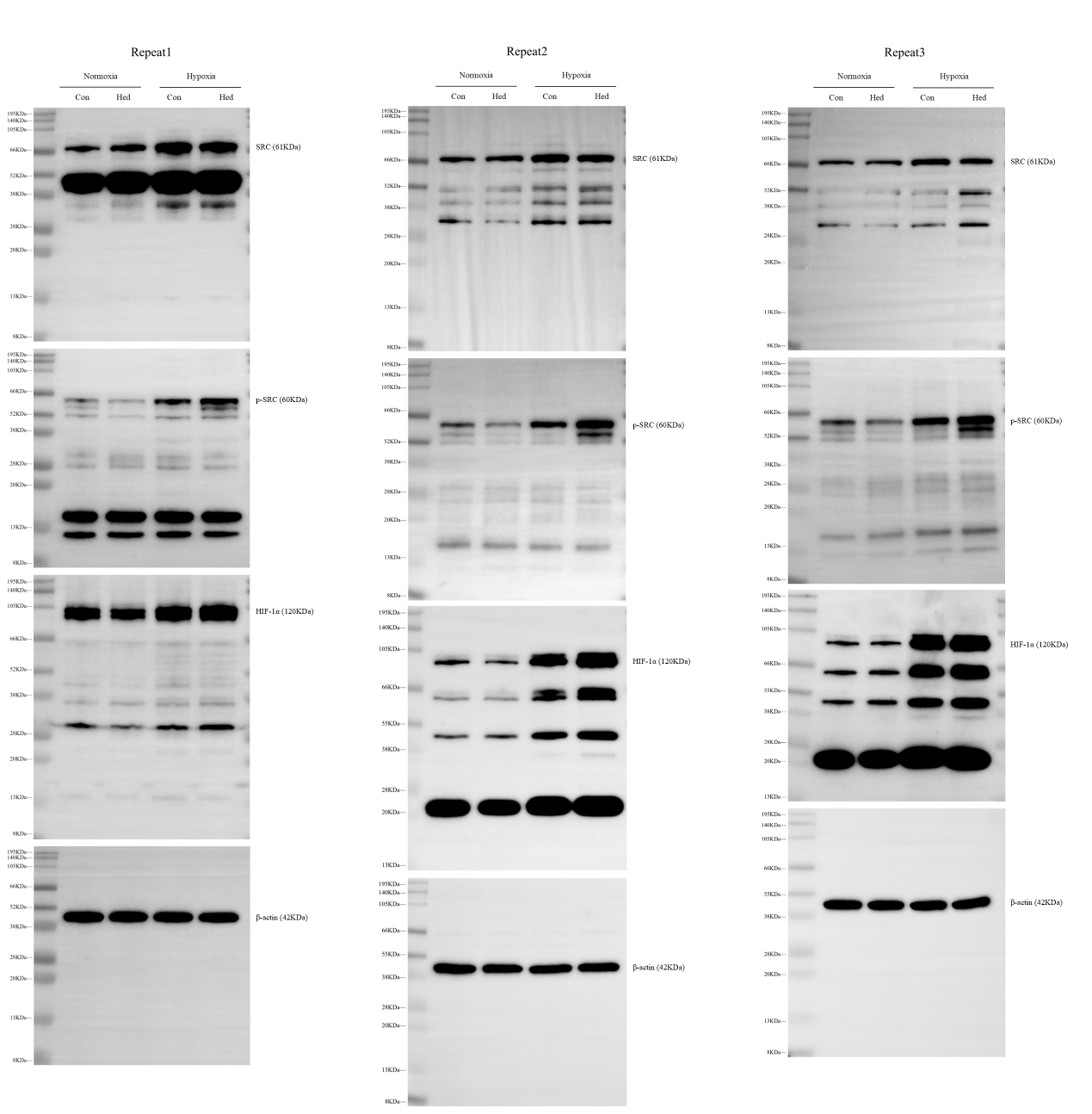


Figure S3. Original Western blot of STAT3, p-STAT3, AKT1.


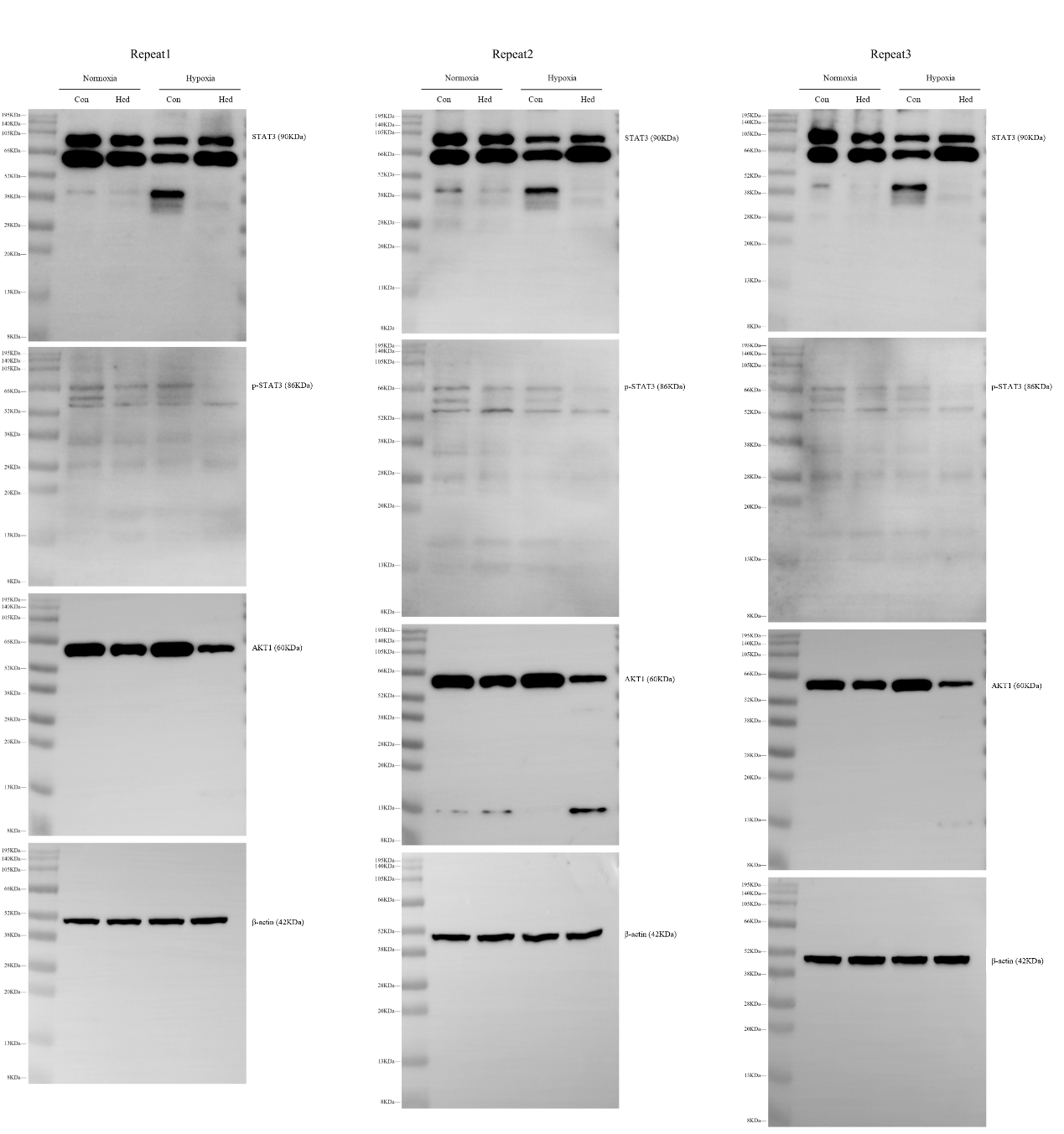

Supplement: Supplementary file 1 — pr5c00022_si_001.zip [file pr5c00022_si_001.zip › Supplementary materials/Figure S1-3. original western blot.docx]
